# Occupational Silica Exposure and Pericarditis: An Uncommon Link

**DOI:** 10.7759/cureus.33856

**Published:** 2023-01-17

**Authors:** Aung Hein, Robert Willington, Yin Wai, Mahima Charan

**Affiliations:** 1 Department of Cardiology, University Hospitals Dorset, Bournemouth, GBR; 2 Department of Internal Medicine, University Hospitals Dorset, Bournemouth, GBR; 3 Department of Respiratory Medicine, Poole General Hospital, Poole, GBR; 4 Health Education and Rheumatology, University Hospital Derby and Burton NHS Trust, Derby, GBR

**Keywords:** echocardiography, vasculitis, granulomatosis with polyangiitis, silica exposure, pericarditis

## Abstract

Granulomatosis with polyangiitis (GPA) is a multi-system necrotising vasculitis, particularly affecting small vessels. Upper respiratory tracts, lungs and kidneys are common target organs, while cardiac involvement would also be the first and rare manifestation of the disease. In GPA with cardiac involvement, structures such as the pericardium, myocardium, endocardium and conduction system could be involved. In the literature, there are reports of an association between autoimmune diseases and silica exposure. In our case, a 73-year-old sculptor with regular exposure to silica presented with pericarditis and was later diagnosed with granulomatosis with polyangiitis. This report provides additional evidence of an association between silica exposure and autoimmune vasculitis.

## Introduction

Granulomatosis with polyangiitis (GPA, formerly Wegener’s granulomatosis) is a multi-system disease that can be described as a necrotizing vasculitis characterised by granulomatous infiltrates [1]. It commonly affects the respiratory tract and kidneys, causing necrotising glomerulonephritis [2]. Coronary vasculitis and pericarditis are the commonest presentations in up to 50% of cases of GPA with cardiac involvement [3]. GPA with cardiac involvement carries an overall mortality rate between 15% and 45% [4]; therefore, it is vital that physicians are aware of the variety of possible clinical presentations, both early and late, so that appropriate investigations and management can be initiated in a timely manner. GPA is often a diagnostic challenge with a wide variety of presentations. Cardiac involvement in small to medium-sized vasculitis is well-documented and a common presentation (5.7%) in patients with antineutrophil cytoplasmic antibodies (ANCA)-positive vasculitis such as GPA [5]. It remains unclear whether cardiac involvement is associated with a worse prognosis in GPA, with studies reaching differing conclusions [5-8]. The cardiac structures involved may include the pericardium, valves, conductive system, myocardium, or endocardium [6]. It is less widely recognised that occupational risk factors, such as silica exposure, may increase the risk of developing ANCA-associated vasculitides [9].

## Case presentation

**Figure 1:**
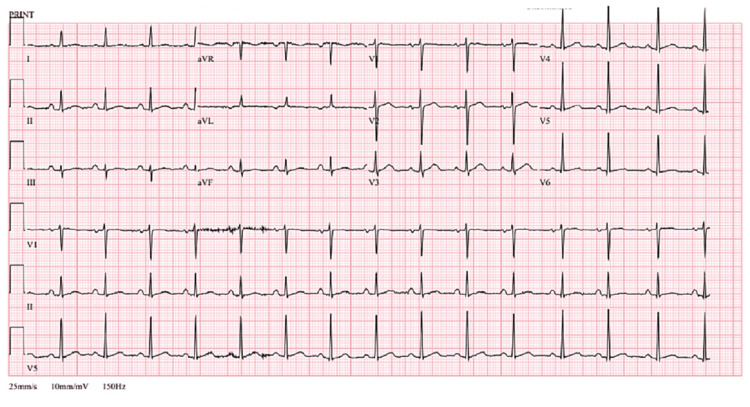
ECG ECG showed sinus rhythm without ST segment or T wave changes

**Figure 2:**
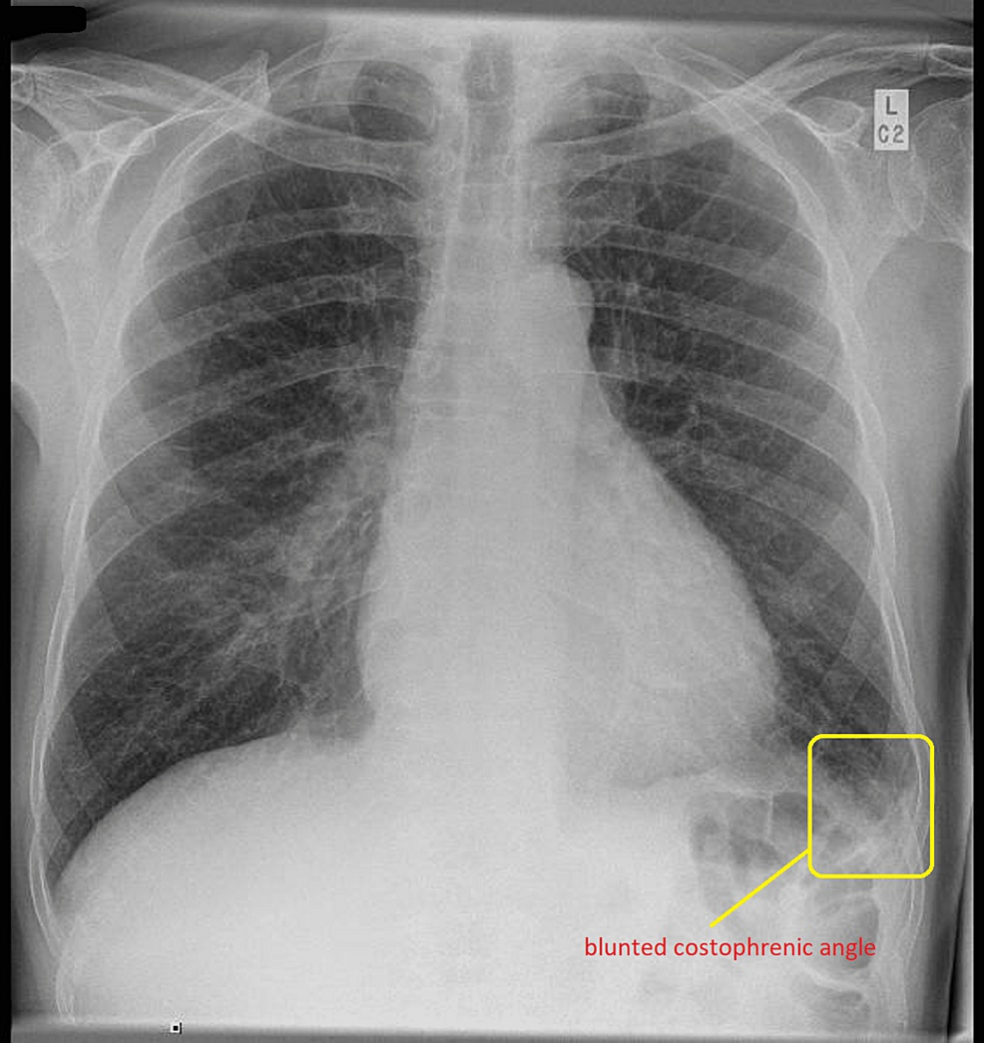
Chest X-ray Chest X-ray showing mildly blunted left costophrenic angle suggestive of small pleural effusion

A 73-year-old man was admitted after a collapse at home, which was associated with a four- to six-week history of intermittent retrosternal chest pain. Other symptoms included poor appetite, fatigue, night sweats, and several falls without loss of consciousness. He was an active smoker with a 40-pack-year history, and his weekly alcohol consumption was approximately 15 to 20 units per week. He denied other respiratory, abdominal, or genito-urinary symptoms. He lived with his wife and, through his occupation as a sculptor, had substantial ongoing exposure to silica dust. On examination, his temperature was 36.4 degrees centigrade, blood pressure 133/67 mmHg, pulse rate 112 bpm, respiratory rate 20 per minute, and oxygen saturation was 96% on air. He had normal heart sounds and no signs of heart failure. There was no rash or joint pain. His neurological examination was normal. His ECG showed sinus rhythm with a PR interval of 156 ms, QRS of 70 ms, QTc of 446 ms and rate of 99 beats per minute without ST segment and T wave changes (Figure 1). Chest X-ray showed clear lung fields with a small, left left-sided pleural effusion (Figure 2). Blood tests revealed normocytic anaemia, significantly raised C-reactive protein (CRP; 120 mg/L), ESR (erythrocyte sedimentation rate; 72 mm/hr), and D-dimer (2169 ng/ml), but normal renal function as estimated by creatinine (89 umol/L) and troponin-T was negative. Anti-proteinase 3 (pr3 antibody) subsequently returned significantly raised at 134 IU/ml. Given the high inflammatory markers, we sent blood culture and coronavirus disease 2019 (COVID-19) PCR tests, which were negative. With pleural effusion, we sent a sputum sample for microbiology study, which only showed normal upper respiratory tract flora. His procalcitonin level was borderline and did not support bacterial infection either. See Table 1 for all the blood tests.

**Table 1:** Full blood count, clotting screen, C-reactive protein, procalcitonin, renal function, liver function test, connective tissue disease screen, iron study and D-dimer Connective tissue disease screen contains dsDNA, Sm, Rib-P, PNCA, U1-snRNP, Ro, La, Scl-70, CENP, Fibrillarin, RNA Polymerase III, Jo-1, Mi-2 and PM-Scl. FU, follow-up

Days	day1	day2	day3	day4	day5	day6	FU1	FU2	FU3	FU4	Reference range
Haematocrit (L/L)	0.31	0.33	0.32	0.31	0.3	0.38	0.44 0.44	0.46 0.46	0.45	0.47 0.47 0.47	0.40 - 0.50
Lymphocyte count (10*9/L)	1.1	1.2	1.6	1.5	2.2	0.6	1.2 1.2	1.3 1.3	0.9	0.8 0.8 0.8	1.0 - 3.0
Erythrocyte sedimentation rate (mm/h)	72		120			11	9 9	2 2	2	2	1.0-30.0
Neutrophil count (10*9/L)	7.2	8.8	8.1	16.9	12.6	17.9	18.1 18.1	13.1 13.1	12.8	11.9 11.9 11.9	2.0 - 7.0
Haemoglobin estimation (g/L)	102	105	101	97	94	114	136 136	144 144	145	154 154 154	130 - 170
Platelet count (10*9/L)	464	473	464	531	489	319	247 247	226 226	209	210 210 210	150 - 410
Mean corpuscular volume (MCV) (fL)	87	88	91	91	91	96	98 98	96 96	96	96 96 96	83 - 101
Total white cell count (10*9/L)	9.4	11	11	18.9	15.4	18.8	19.7 19.7	14.9 14.9			4.0 - 10.0
Activated partial thromboplastin time* (*APTT) ratio	1.2	1.25	1.22	1.08 1.08							0.85 - 1.15
International normalised ratio	1.4	1.5	1.49	1.48 1.48							0.90 - 1.10
Prothrombin time (s)	15.9	17	16.8	16.70 16.70							10.20 - 12.60
C-reactive protein (CRP)	120	117	110	116	119	65	14	2.3			0 - 9
Procalcitonin (ng/mL)	0.1										<0.1
Serum creatinine (µmol/L)	78	77	70	71	80	86	90	81	92		59 - 104
Glomerular filtration rate (GFR) calculated abbreviated MDRD (mL/min/1.73m*2)	85	86	90	90	82	75	72	82	70		-
Serum potassium (mmol/L)	4.7	5.1	4.2	4.6	4.2	4.2	4.1	4.2	4.5		3.5 - 5.0
Serum sodium (mmol/L)	137	134	137	137	137	141	140	141	140		132 - 146
Serum urea level (mmol/L)	4.1	8.2	8.3	9.5	8.2	6.5	6	5.5	5.5		2.5 - 6.7
Serum albumin (g/L)	29	30	33	39	41		41 41 41	45 45			35 - 48
Serum alkaline phosphatase (U/L)	77	80	88	79	56 56	44 44	54 54 54	61 61			30 - 150
Serum alanine transaminase (ALT) level (U/L)	17	15	15	19	17 17	54 54	20 20 20	20 20			0 - 35
Serum total bilirubin level (µmol/L)						18 18	5 5 5	4 4			0 - 17
Serum calcium (mmol/L)	2.25	2.29		2.4							2.20 - 2.60
Corrected serum calcium level (mmol/L)	2.46	2.48		2.47							2.20 - 2.60
Serum iron (µmol/L)	4										11.0-25.0
Serum total iron binding capacity (µmol/L)	36										45.0-72.0
Transferrin saturation index (%)	11										
Connective tissue disease screen (ratio)	0.2										0.0-0.6
Myeloperoxidase antibody (IU/ml)	0.2										0.00-3.40
Proteinase 3 antibody (IU/ml)	134										0.00-1.90
D-dimer (ng/mL)	2169										0.0-243.0
Troponin (ng/L)	<14										<14

**Figure 3:**
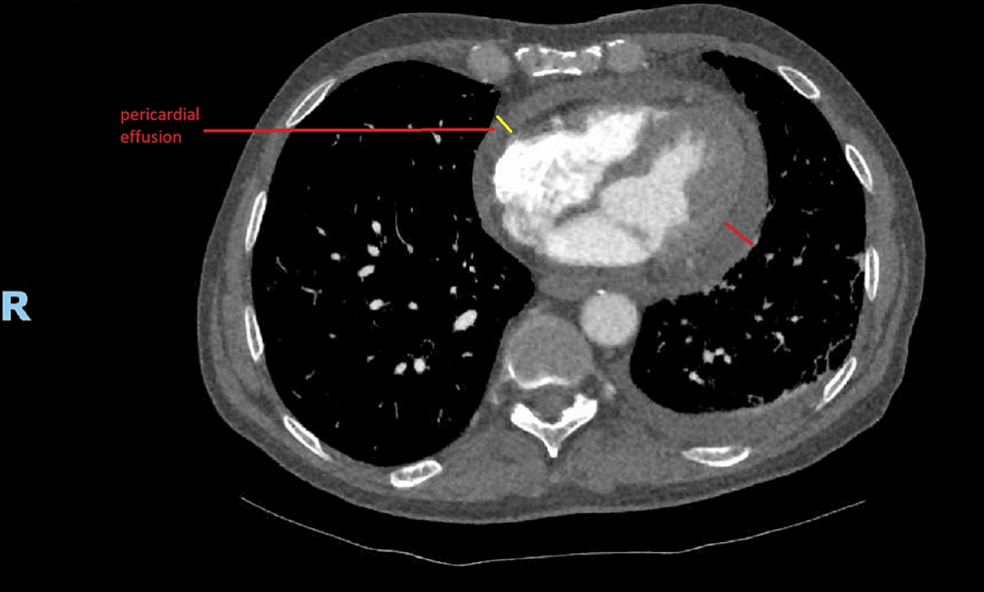
CT pulmonary angiogram CT pulmonary angiogram showing pericardial effusion

**Figure 4:**
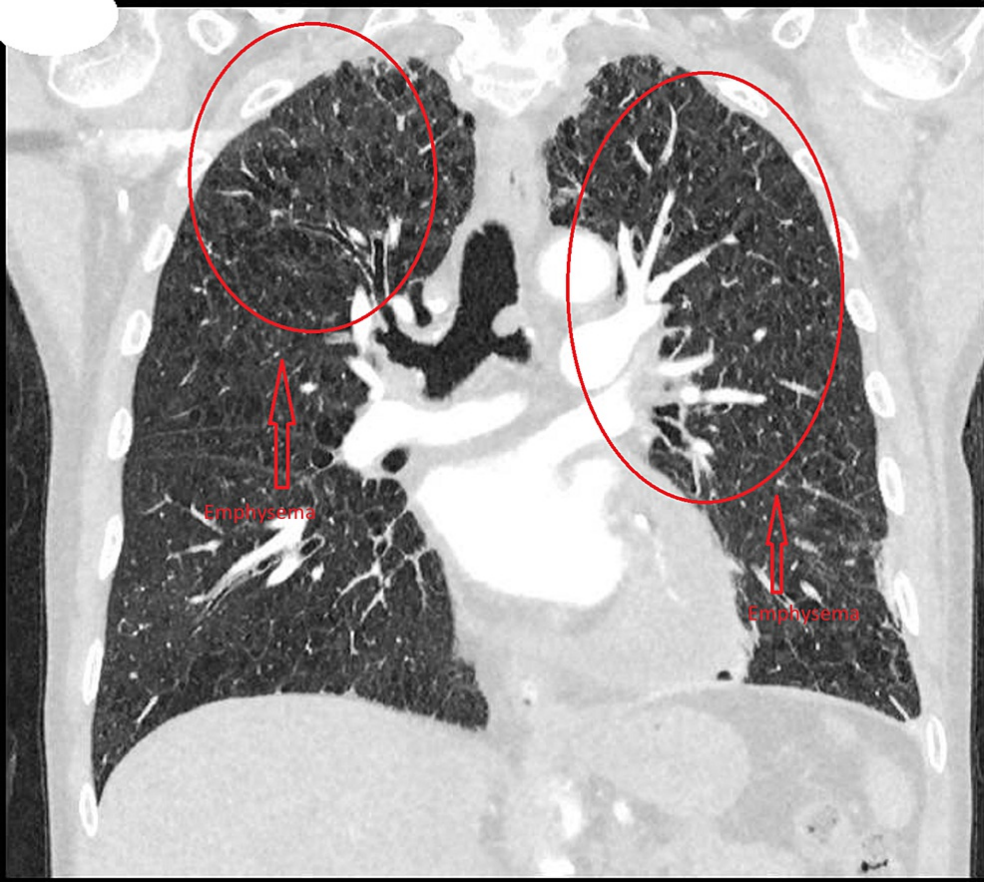
CT pulmonary angiogram CT pulmonary angiogram showing emphysema and no pulmonary embolus

A CT pulmonary angiogram (CTPA) requested in light of the chest pain and raised D-dimer revealed emphysema, bi-apical scarring, pericardial thickening, and pericardial effusion but effectively excluded pulmonary embolism (Figures 3, 4).

**Figure 5:**
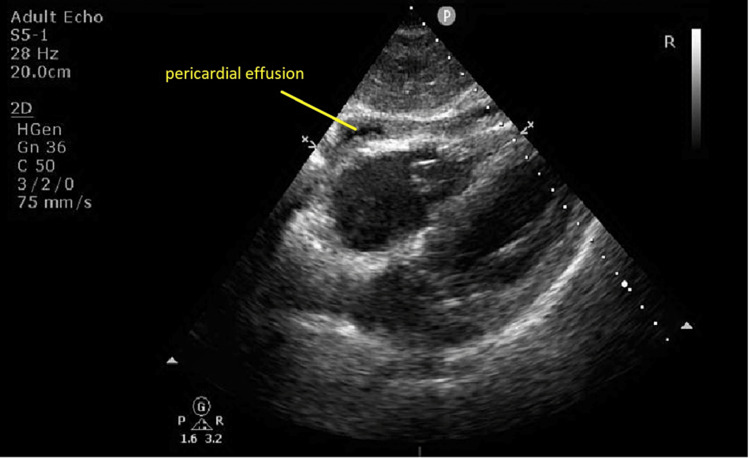
Transthoracic echocardiography: subcostal view Subcostal view of echocardiography showing pericardial effusion

Departmental echocardiography was reported as normal left ventricular cavity size with hypokinetic inferolateral wall, basal-mid anterolateral and basal inferior wall segments, small pericardial effusion with impaired overall LV systolic function with an ejection fraction of 50% (Figure 5).

Since his clinical picture and blood results strongly suggested that he had ANCA-associated vasculitis, he was referred to rheumatology and given pulsed methylprednisolone. Intravenous methylprednisolone 500 mg was given for three days and then stepped down to oral prednisolone initially 40 mg for two weeks with urgent rheumatology follow-up. Colchicine was continued given ongoing chest pain. Symptoms, inflammatory markers, and normocytic anaemia subsequently improved. He was later started on methotrexate and steroids were weaned down with the intention to stop. He was regularly followed up with repeat blood tests, which all showed that his vasculitis was under control.

## Discussion

As a multi-system disease, granulomatosis with polyangiitis can present with various symptoms. In our case, the presentation was with collapse and chest pain. With high D-dimer, clinicians initially needed to rule out pulmonary embolism. Other routine differentials would be musculoskeletal chest pain or lower respiratory tract infection. After pulmonary embolism was ruled out, we saw effusion on the CT scan. With regard to the diagnosis of pericarditis, we referred to the guidelines from the European Society of Cardiology released in 2015 [10]. The European Society of Cardiology set out diagnostic criteria for pericarditis, including pericarditic chest pain, pericardial rub, new widespread ST elevation or PR depression, and pericardial effusion. The patient did not report classical pericarditic chest pain. However, he had subacute intermittent chest pain and pericardial effusion, which was suggestive of pericardial inflammation. With intermittent chest pain and pericardial effusion, we concluded that the patient had pericarditis secondary to GPA.

This presentation might lead to misdiagnosing viral pericarditis, commonly seen in emergency departments or acute medical units. It became clear upon the detailed history that the patient had chronic inflammatory symptoms such as fatigue, lethargy, night sweats and joint pain. This detailed history widened our differential of possible underlying auto-immune disease and ANCA-associated vasculitis. In addition, when we took an occupational history, the association of regular silica exposure helped us to think laterally. The literature reported that auto-immune diseases were also linked to silica exposure [11,12], and in our case, the patient has had silica exposure for several years as a sculptor. It led us to consider the previously reported associations between silica dust exposure and autoimmune disease. After we had ruled out other possible differential diagnoses, we concluded that our case of pericarditis was secondary to ANCA-associated vasculitis. There would be a possible association between his occupational exposure to silica and his auto-immune disease. We consulted him regarding his occupational risk and provided our opinion.

## Conclusions

In acute admission units, it is not uncommon to see cases presented with pericarditis. Since viral infections are common causes of pericarditis, we could overlook the auto-immune aetiology with pericarditis as the initial manifestation. Careful symptom analysis and thorough history-taking paved the way to getting the correct diagnosis in time. In addition, it has been reported that silica exposure is a recognised risk factor for auto-immune diseases, and our case would provide additional evidence for this association. We learned from the case that clinicians should consider auto-immune screening when the cause of pericarditis or pericardial effusion is unclear and occupational history, particularly silica dust, is important in cases of auto-immune diseases.

## References

[1] Miłkowska-Dymanowska J, Laskowska P, Rzuczkowski M (2019). Untypical manifestations of granulomatosis with polyangiitis—a review of the literature. SN Compr Clin Med.

[2] Kubaisi B, Abu Samra K, Foster CS (2016). Granulomatosis with polyangiitis (Wegener's disease): an updated review of ocular disease manifestations. Intractable Rare Dis Res.

[3] Florian A, Slavich M, Blockmans D, Dymarkowski S, Bogaert J (2011). Cardiac involvement in granulomatosis with polyangiitis (Wegener granulomatosis). Circulation.

[4] Safak O, Gursul E, Polat M (2016). Wegener’s granulomatosis with cardiac involvement. Int J Cardiovasc Acad.

[5] McGeoch L, Carette S, Cuthbertson D (2015). Cardiac involvement in granulomatosis with polyangiitis. J Rheumatol.

[6] Guillevin L, Pagnoux C, Seror R, Mahr A, Mouthon L, Toumelin PL (2011). The Five-Factor Score revisited: assessment of prognoses of systemic necrotizing vasculitides based on the French Vasculitis Study Group (FVSG) cohort. Medicine (Baltimore).

[7] Goodfield NE, Bhandari S, Plant WD, Morley-Davies A, Sutherland GR (1995). Cardiac involvement in Wegener's granulomatosis. Br Heart J.

[8] Hara A, Wada T, Sada KE (2018). Risk factors for relapse of antineutrophil cytoplasmic antibody-associated vasculitis in Japan: a nationwide, prospective cohort study. J Rheumatol.

[9] Gómez-Puerta JA, Gedmintas L, Costenbader KH (2013). The association between silica exposure and development of ANCA-associated vasculitis: systematic review and meta-analysis. Autoimmun Rev.

[10] Adler Y, Charron P, Imazio M (2015). 2015 ESC guidelines for the diagnosis and management of pericardial diseases: the task force for the diagnosis and management of pericardial diseases of the European Society of Cardiology (ESC). Endorsed by: the European Association for Cardio-Thoracic Surgery (EACTS). Eur Heart J.

[11] Steenland K, Goldsmith DF (1995). Silica exposure and autoimmune diseases. Am J Ind Med.

[12] Janssen LM, Ghosh M, Lemaire F, Michael Pollard K, Hoet PH (2022). Exposure to silicates and systemic autoimmune-related outcomes in rodents: a systematic review. Part Fibre Toxicol.

